# The Arrival of the Frequent: How Bias in Genotype-Phenotype Maps Can Steer Populations to Local Optima

**DOI:** 10.1371/journal.pone.0086635

**Published:** 2014-02-05

**Authors:** Steffen Schaper, Ard A. Louis

**Affiliations:** 1 Rudolf Peierls Centre for Theoretical Physics, University of Oxford, Oxford, United Kingdom; Fred Hutchinson Cancer Research Center, United States of America

## Abstract

Genotype-phenotype (GP) maps specify how the random mutations that change genotypes generate variation by altering phenotypes, which, in turn, can trigger selection. Many GP maps share the following general properties: 1) The total number of genotypes 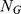 is much larger than the number of selectable phenotypes; 2) Neutral exploration changes the variation that is accessible to the population; 3) The distribution of phenotype frequencies 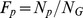, with 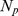 the number of genotypes mapping onto phenotype 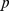, is highly biased: the majority of genotypes map to only a small minority of the phenotypes. Here we explore how these properties affect the evolutionary dynamics of haploid Wright-Fisher models that are coupled to a random GP map or to a more complex RNA sequence to secondary structure map. For both maps the probability of a mutation leading to a phenotype 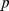 scales to first order as 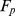, although for the RNA map there are further correlations as well. By using mean-field theory, supported by computer simulations, we show that the discovery time 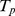 of a phenotype 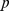 similarly scales to first order as 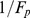 for a wide range of population sizes and mutation rates in both the monomorphic and polymorphic regimes. These differences in the rate at which variation arises can vary over many orders of magnitude. Phenotypic variation with a larger 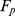 is therefore be much more likely to arise than variation with a small 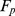. We show, using the RNA model, that frequent phenotypes (with larger 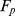) can fix in a population even when alternative, but less frequent, phenotypes with much higher fitness are potentially accessible. In other words, if the fittest never ‘arrive’ on the timescales of evolutionary change, then they can't fix. We call this highly non-ergodic effect the ‘arrival of the frequent’.

## Introduction

Darwin's account of biological evolution [1] stressed the importance of natural selection: If some individuals are better adapted to their environment than their competitors, their offspring will come to dominate the population. The fittest survive and the less fit go extinct. Yet selection alone is not sufficient to drive evolution because natural selection reduces the very variation that it requires to operate. It was only recognised well after Darwin's day [2], in part through the success of the Modern Synthesis, that the fuel for selection is provided by mutations that make offspring genetically different from their parents. Crucially, mutations change genetically stored information (the *genotype*) while selection operates on the physical expression of this information (the *phenotype*). Understanding the relation between genotypes and phenotypes – the GP map – is therefore crucial to understanding evolutionary dynamics [3].

GP mappings have been studied at different levels of abstraction [4] The most basic systems are concerned with the sequence-to-structure(-to-function) relation of single molecules such as RNA [5] or proteins [6]–[8], but higher-level systems such as protein complexes [9], gene-regulatory networks [10] and developmental networks [11] have also been studied. Even though these GP maps arise in quite different contexts, they share several interesting properties:

1. Most basically, the number of possible genotypes 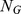 is typically much greater than the number of possible phenotypes 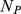, so the map is many-to-one. As a consequence, many mutations may conserve the phenotype, leading to mutational robustness. Important prior work has linked such robustness to the concept of neutral spaces, namely the set of all genotypes that map to a particular phenotype, with the additional property that they be linked by neutral mutations [4], [5], [12].
2. Even though 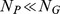, the accessible genetic neighbourhood of a single genotype 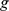 that generates a given phenotype 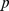 may include significantly fewer alternative phenotypes (potential variation) than is found in the neighbourhood of the (neutral) set 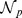 of all 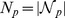 genotypes that map onto phenotype 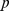. Exploration of a neutral space can therefore increase the variety of phenotypes discovered by a population [13], [14].
3. Perhaps the most striking commonality of these GP maps is a strong bias in assignment of genotypes to phenotypes: Most phenotypes are realised by a tiny proportion of all genotypes, while most genotypes map into a small fraction of all phenotypes. This property is shared by all the GP maps we noted before. Typically the number 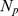 of genotypes per phenotype 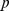 and the related phenotype frequencies 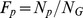 can vary over many orders of magnitude. Such huge variations are likely to have an effect on the course of evolution.

In this paper we study the evolutionary dynamics of a classical Wright-Fisher model, but with explicit microscopic GP maps that capture the three generic properties of such maps introduced above. Motivated by the strong bias in the distribution of the 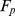 observed for many GP maps, we derive a mean-field like approximation for the average probability 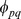 that a mutation will change a genotype that generates phenotype 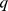 into one that generates phenotype 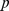. This approximation greatly simplifies the dynamics, allowing us to calculate analytic expressions for quantities such as the median time 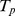 for phenotype 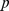 to first appear in the population as a function of population size 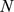, the point mutation rate 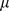, genome length 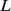 and the mutation probabilities 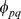.

These approximations are then tested against extensive simulations of two models: firstly, a simple GP map where the genotypes are randomly assigned to phenotypes according to a pre-determined distribution for the frequencies 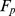 and secondly, the well-known mapping of RNA sequence to secondary structure [4], [5], [15], which is more complex, but also more biologically realistic. We focus on the case where a population of 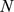 individuals has initially equilibrated at a fitness maximum given by phenotype 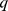, and then measure the median time 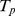 for alternative phenotype 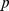 to first arise in the population.

Our analytic expressions agree quantitatively with the simulations in the polymorphic limit where 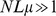, and also in the opposite monomorphic limit 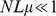. In between these regimes a single scaling factor must be included. In all regimes the median discovery time 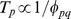. For the random model 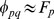; this scaling also holds for the more complex RNA mapping, although there is significantly more scatter due to local correlations within the neutral spaces and for some phenotypes we find 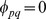 even though 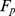 is large (this can be due to biophysical constraints explained for example in ref. [16]). Despite such higher order effects, the variation of the 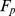 over many orders translates directly into the 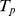. More frequent (higher 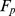) phenotypes are therefore discovered more rapidly and more often along evolutionary trajectories. In this way the structure of the GP map can play a key role in determining evolutionary outcomes.

Finally, we employ the RNA GP map to study the case where two phenotypes 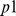 and 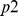 are both more fit than the source phenotype 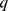, but where 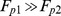 (or more accurately 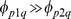). Direct simulations show that phenotype 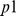, which is more frequent, is much more likely to fix in the population, even if its fitness is much lower than that of 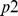, an effect we call ‘the arrival of the frequent’.

## Results

### Theoretical framework

We study the evolution of a population of 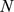 asexual haploid individuals. Each individual 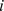 carries a genotype 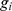 of 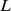 letters taken from an alphabet of size 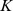. The individual's phenotype 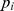 is determined from 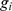 via the GP map. The population evolves in in discrete, non-overlapping generations according to the classical Wright-Fisher model for haploid individuals: At each generation 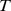, 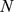 parents are drawn with replacement with probability proportional to their fitness 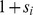 with the constraint that the population size (or carrying capacity) 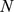 is fixed. Each parent gives rise to one offspring, and the offspring make up the population for the next generation. During reproduction, each base in the genotype of length 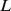 mutates to a random alternative base with probability 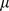. The number of mutations (that is, the Hamming distance) 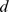 between parent and offspring is thus distributed binomially according to 
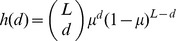
. In this way the set 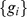 of 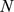 genotypes changes at each generation.

The expected number of individuals with phenotype 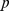 that arises at generation 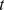 can be written as:
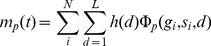
(1)where 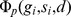 is the probability that a 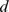 fold mutation of genotype 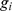 (selected for reproduction according to fitness 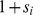) generates an individual with phenotype 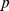. It takes into account the mutational connections between the 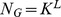 genotypes that make up the GP map. The probability of not finding 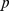 is approximately given by the Poisson distribution as 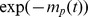.

**Figure 1:**
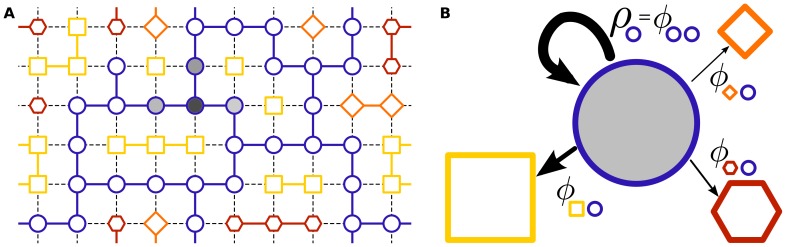
Illustration of the mean field approximation. *A*) An example genotype space: Each point corresponds to a unique genotype; shape and color of the marker indicate the phenotype. Genotypes joined by edges can be interconverted by single mutations. Edges for neutral mutations share the color of the (conserved) phenotype, non-neutral mutations are shown as black dashed lines. The shading of the genotypes illustrates the number of individuals carrying the respective genotype in a hypothetical population. The mutations away from the genotypes occupied by the population determine the accessible phenotypes. *B*) Our meanfield approximation averages over the internal structure of neutral spaces. So neutral spaces are represented by the markers of their phenotypes only, with the size representing the neutral space size (ie. number of genotypes in the space). The uniform shading of the blue neutral space implies that in the meanfield approximation, the population is assumed to continually explore the neighbourhood of its entire neutral space. Mutational outcomes are thus determined from the local frequencies of phenotypes around the neutral space, as measured by the 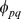 coefficients. This mean field approximation allows us to derive analytic forms that can be compared to simulations of the full GP map.

While exact, these dynamic expressions depend implicitly on time through stochastic changes in the set 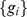, and are typically very hard to solve. In order to gain intuitive insight, we employ a number of simplifications and approximations, motivated in part by the general properties of GP maps discussed in the introduction. First, we assume that 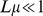 so that for 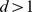, 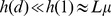, which means that we can ignore higher order mutations (terms with 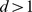 in Eq. (1)). For a given source phenotype 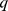 (where the fitnesses of all genotypes mapping into 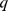 are equal, and so we take 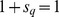 for simplicity) we can then calculate the mean probability 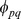 that a single point mutation will generate another phenotype 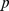:
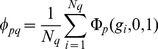
(2)where the sum is over the set 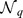 of all 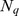 genotypes that generate phenotype 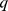 (see also Figure 1). It is convenient to introduce the robustness of phenotype 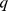 as the average probability over all 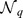 of neutral mutations: 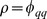. If we consider the case where at generation 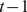 the whole population is on 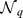, then Eq. (1) simplifies in this mean-field (or pre-averaged) approximation to:
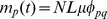
(3)


#### The polymorphic limit

If 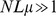 then the population naturally spreads over different genotypes, a regime called the polymorphic limit. Consider the case where 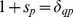 so that the population remains on 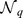, which is one way to model neutral exploration. In the mean-field approximation the expected number of individuals with phenotype 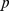 produced per generation is now independent of time, and given by Eq.(3), as long as double mutations can be ignored. The time 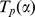 when on average the probability of having discovered 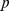 is 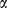 (so that the median discovery time of 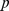 is 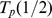) is then given by:
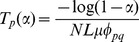
(4)


Eqns. (3–4) should provide a good approximation of the full dynamics in the limit that 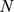 is large enough that variations between individual genotypes 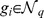 are averaged out, in other words, for the case where the 1-mutant neighbourhood of the population is similar to that of the whole neutral space.

#### The monomorphic limit

Neutral spaces can be astronomically large [17], much bigger than even the largest viral or bacterial populations. In that case, the local neighborhood of the population may not be fully representative of the neighborhood of the entire space. This scenario can most easily be understood in the monomorphic limit where mutants are rare, 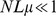, and exploration is dominated by genetic drift. Every neutral mutant has a probability of 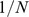 to go to fixation, allowing the population to move to a new genotype. Thus the timescale of fixations is Kimura's famous result [18] 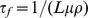, where the robustness 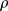 is the probability that a mutation is neutral, so that 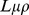 is the rate of neutral mutations.

Between fixations, the population undergoes periods of genotypic stasis in which only the 1-mutant neighborhood of the current genotype 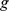 is explored by (rare) mutations. As there are 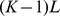 adjacent genotypes, the timescale of this exploration is 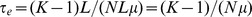.

It is instructive to compare the ratio 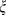 of these two time-scales, defined via
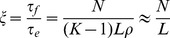
(5)We can use this dimensionless ratio to distinguish between different dynamic regimes. If 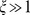, fixation takes much longer than exploration. If we define 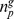 as the number of local neighbours of the genotype 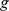 mapping to phenotype 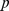 for the current population, then in this limit, phenotypes with 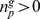 are produced continuously (on a time-scale given by 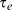) until the population moves to a different genotype. The dynamics under strong genetic drift therefore induce short-term correlations in the mutant phenotypes. Since 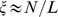, we call this regime the large population limit.

In the opposite extreme 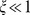, which we call the large genome limit, the population typically moves to a different genotype before all accessible mutants have been explored. In this regime, we do not expect short-term correlations in the mutant phenotypes, simply because every mutant occurs only very rarely.

Actual discovery and neutral fixation times can show strong fluctuations. As our evolutionary process is a Markov process – the next set of mutants depends only on the parents, not on earlier mutants – the first discovery time of a neighbour genotype as well as the arrival time of the neutral mutant “destined” to be fixed, are distributed geometrically (or exponentially in a model with continuous time). Thus the mean of 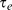 or 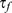 is equal to the respective standard deviation, and any particular evolutionary trajectory can be very different from the average behaviour.

Let 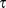 be the actual time the population stays at the current genotype. In the continuous time approximation, 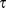 is distributed exponentially with mean 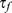. If the genotype 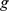 has 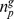 mutations leading to 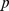 then the probability that 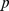 is found during this time is 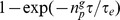. Integrating over the distribution of 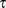, we have the probability 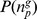 that phenotype 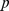 is discovered before the next neutral fixation:
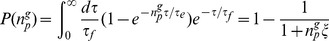
(6)


If fixations are the rate-limiting step (ie. 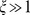), 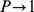 if 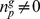, as each neighborhood is searched exhaustively before the population moves on. On the other hand, if fixation is faster than exploration (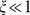), the introduction of alternative phenotypes is determined by random fluctuations, as most available mutants are not produced. To leading order, we find 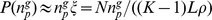. We note that the inverse dependence on 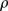 arises from 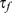: More robust neutral spaces are explored faster, but therefore less thoroughly.

The dynamics in the monomorphic regime are thus relatively straightforward. But whether some new phenotype 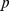 is discovered still depends on the structure of the neutral space which in turn determines how the available phenotypes change upon a neutral fixation. To describe this structure, we turn again to a mean-field approximation: The mutational neighborhood of each particular genotype 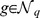 resembles the average over 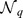. As the mean number of mutations per genotype leading to 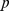 is given by 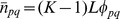, the probability that 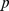 is accessible after a neutral fixation is 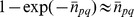 (the approximation is valid provided 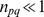, that is 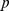 is not accessible from every genotype in the source neutral space; of course, this is just the condition we are interested in, as otherwise neutral exploration would not typically be necessary for phenotype 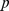 to arise).

Over a large number of generations (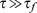), a monomorphic population explores its neutral space uniformly [19]. Assuming that 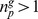 can be ignored in practice, we have 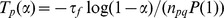. The first discovery time in the large population limit becomes:
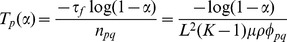
(7)whereas in the large genome limit we obtain
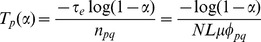
(8)which has the same form as the polymorphic limit, Eq. (4): When the population is too small (compared to the genome length), the exploration of each genotype's mutational neighborhood is typically incomplete. Then, just as in the polymorphic limit, only random fluctuations determine which accessible genotypes are actually realized by the population.

Finally, let us compare our results for large populations in the monomorphic and polymorphic limits. Most importantly, in both cases 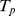 is inversely proportional to 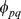: Rare phenotypes are hard to find. Comparing Equations (4) and (7), the only difference is that 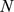 in the polymorphic regime is replaced by 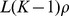 in the monomorphic limit. This difference is intuitive: When the population is diverse, every new individual helps exploration and reduces discovery times. But if all individuals have the same genotype, simply having “more of the same” does not make neutral exploration faster. However, repeated mutants may influence the fixation of adaptive phenotypes.

These results suggest that for intermediate 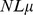 there should be a smooth transition between these two regimes. To quantify the crossover we introduce a factor 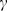 that multiplies 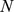 in Eq.(4); we expect that 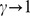 as either 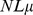 becomes very large (the polymorphic limit) or 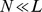 (the large genome limit), and that 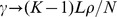 as 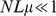 and 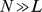 (the large population monomorphic limit).

### Simulations in model GP maps

In order to test our mean-field theory we study two kinds of GP maps that both include the generic properties of GP maps that we introduced earlier.

#### Random GP map

In the random GP map, the total number of phenotypes 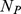 and the frequencies 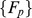 can be set arbitrarily (subject to the normalization constraint 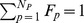). The 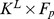 genotypes mapping into phenotype 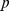 are distributed randomly in genotype space. The statistical properties of the map are thus determined by the parameters 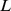, 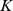, and the set 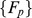.

Studying this map has two motivations: First, ignoring some biophysical detail may help illuminate generic features shared by the systems described in the introduction. Second, a simple model may clarify which deviations from our theory arise from population dynamic effects rather than from detailed (and system-specific) structure in the GP map.

In this simple model, correlations between genotypes are absent, facilitating analysis of the resulting neutral spaces. For example, 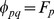 is a good approximation as long as 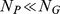 and 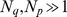. Also, there is a percolation threshold 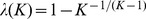: thus only phenotypes with 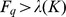 have completely connected neutral spaces [20].

Here we study a particular random GP map with 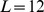, and 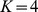 (as in DNA and RNA) so that there are 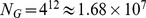 genotypes. These map onto 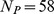 phenotypes distributed with frequencies 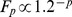. The 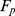 vary over about 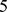 orders of magnitude, a range similar to the 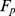 of 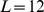 RNA (see also Figure S1). To make sure that the largest neutral space percolates, its frequency is set separately as 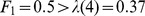. For several values of 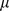, we simulated 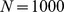 individuals for up to 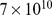 generations. The fitness was set as 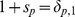 so that we are effectively modelling neutral exploration on the space 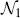, which is convenient for measuring all 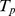. We measured first discovery times for the 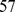 alternative phenotypes over 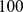 independent simulations to obtain the median time 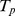.

**Figure 2:**
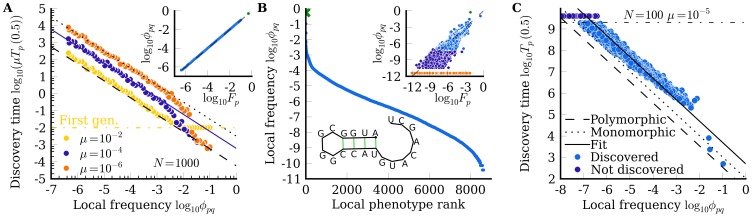
Test of the meanfield model. *A*) Median discovery times 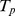 for the random GP map averaged over 100 simulations with 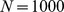 and varying mutation rates. Note that the y-axis is scaled with 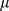. In the the polymorphic limit (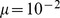), Eq. (4) (dashed line) describes discovery times well for 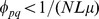. Phenotypes with larger 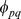 are part of the standing variation typically found in the first generation (yellow dash-dotted line). In the monomorphic limit (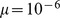), Eq. (7) (dotted line) quantitatively describes 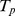 for 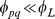, whereas Eq. (4) tracks the simulation data with just one fit parameter 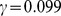 multiplying 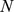 for the intermediate regime with 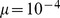 (solid line). For 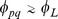 the curves follow Eq. (4), for reasons described in the text. *Inset*: For the random GP map the local phenotype frequency 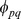 correlates very well with the global frequency 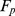. *B*) Local frequency 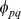 ranked for the 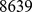 phenotypes that link with single point mutations from the 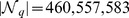 genotypes that map to this RNA structure; an example sequence from 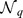 is shown in the figure. *Inset*: The local connections 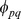 are roughly proportional to the global frequency 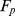, but there is significant scatter due to the internal correlations of the RNA neutral spaces. Organge points depict the 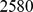 phenotypes for which 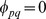. Light blue points depict the 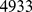 phenotypes that are discovered in our simulations, and the dark blue points depict the 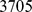 accessible phenotypes that are not found (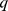 itself is shown in green). *C*) Simulations of 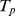 (blue dots) versus 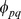 for the RNA phenotype shown in B), compared to Eq. (4) (solid line) with a factor 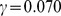 multiplying 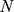. Here 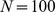, 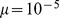 and the simulations were run for 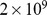 generations. Also shown are the purely polymorphic (dashed) and monomorphic (dotted) predictions. Dark blue dots above 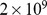 (dot-dashed line) depict some of the 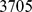 accessible phenotypes that are not found (as can be seen in see the inset of B). We estimate that about 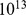 generations would be needed to find the phenotypes with the smallest 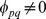.

Figure 2A depicts these median discovery times 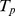 for simulations ranging from the polymorphic regime 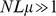 to the monomorphic limit 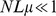 (see also Figure S2). We note the following:

1. For all regimes the 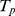 vary over many orders of magnitude, but they are found in fewer generations for larger 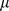.
2. Locally frequent phenotypes (i.e. those with high 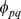) are much easier to discover. The inset of Figure 2A shows that 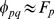, so this conclusion carries over to frequent phenotypes with large 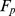.
3. A subset of the phenotypes with 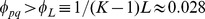 are likely to be in the one-mutation neighbourhood of any genotype. In the monomorphic regime these are are then found by exploration of a genome so that 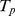 is given by Eq. (8), which has the same form as the polymorphic limit, Eq. (4), as can be seen in Figure 2A. Discovery times cross over to the regime where neutral exploration is required when 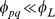. Such behaviour can be viewed as a finite size effect: 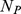 typically increases with 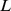. Therefore the largest 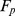 will likely decrease for larger systems, so that a smaller fraction of phenotypes can be found without neutral exploration.
4. In the fully polymorphic regime where each individual essentially explores independently, any phenotype with 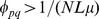 is likely to be part of immediately accessible *standing variation* [21] in the initial population, and is therefore found quickly. Indeed, in Figure 2A for 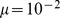, where 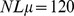, these phenotypes are typically found in one or two generations on average. However, for rarer phenotypes, where neutral exploration is important, the 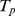 are well approximated by Eq. (4). Again, the fraction of phenotypes that are immediately accessible should decrease for larger 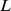.
5. In the intermediate regime 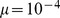, where 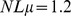, the population spreads over more phenotypes than in the monomorphic regime, but over fewer than in the polymorphic regime. Thus the crossover to the regime where neutral exploration is important occurs at a smaller 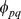 than for the monomorphic regime. In this intermediate 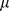 regime neither Eq. (4) nor Eq. (7) suffices. Instead, we use the previously introduced factor 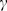 that multiplies 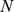 in Eq. (4) to achieve quantitative accuracy. In Appendix S1 and in Figures S3 and S4, we explore the scaling of 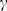 with the parameters 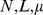, for different dynamic regimes, and for a range of 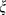.

In summary then, our theory derived in the previous section accurately describes the median discovery time 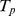 of this simple random GP map as a function of the parameters 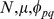. We find that 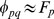, and thus 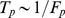 in all regimes studied. The more frequent the phenotype, the earlier (and more often, see Figure S2) it appears as potentially selectable variation in an evolving population. Given the success of our theory for the random model, we now will test our theory and conclusions for a more complex GP map.

#### RNA secondary structure mapping

One of the best studied GP mappings has RNA genotypes of length 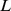 made up of nucleotides G, C, U and A. The phenotypes are the minimum free-energy secondary structures for the sequences, which can be efficiently calculated [15]. The number of genotypes grows as 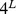, while the number of phenotypes is thought to grow roughly as 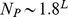 [4] so that 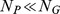. Moreover, sampling and exact enumerations[5], [16], [22] have shown that the distribution of phenotype frequencies 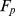 is highly biased, with a small fraction of phenotypes taking up the majority of genotypes. The neutral spaces 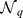 are typically broken up into a number of large components that are connected by single point mutations that allow neutral exploration [16], [22]. By exhaustive enumeration of the 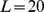 RNA mapping (see also Figure S5) we calculate the 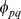 between several neutral components of the 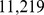 distinct secondary structures that the 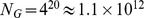 genotypes map to.

Figure 2b shows the 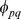 for the largest component of the phenotype 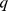 drawn in the figure. This phenotype is ranked as the 3rd most frequent for 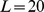 and exhibits behaviour typical of this system. First, the 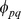 vary over many orders of magnitude. Second, as shown in the inset if 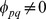, then the *local* 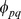 are, to first order, proportional to the *global* 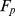. Finally, this neutral space connects to just over 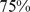 of the total 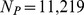 phenotypes in this particular map: Some 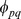 are zero even though 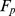 can be quite large. Generally, the number of phenotypes that can be reached from 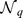 increases with 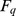 [13], [16].

We performed extensive simulations of the 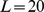 RNA system. Typical results are shown in Figure 2B. *First*, we note that the median discovery times vary over many orders of magnitude. The most frequent are found in a median time of 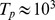 generations while after the maximum measured time of 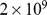 generations, over 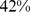 of the directly accessible phenotypes (with 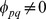) have still not been found. We estimate that over 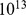 generations would be needed to discover all accessible phenotypes, giving a ten order of magnitude range in the 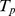. *Second*, the local frequency 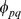 is a good predictor for ranking 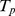 (see Figure S6 for a comparison of 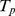 and global frequency 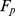). Further, the criterion 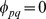 accurately predicts that phenotypes are *not* discovered (see also Figure S6). However, in contrast to the random GP map, the 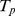 are discovered at a slower rate than predicted by Eq. (7). Instead, we use a single 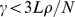 to renormalise 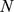 in Eq. (4). This slower discovery rate reflects the internal structure of the RNA: similar genotypes typically have similar mutational neighbourhoods [23], and so the population needs to neutrally explore longer in order to find novelty. Nevertheless, a single 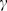 factor yields a remarkably good fit for all the different phenotypes 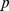 (something we find for all source phenotypes 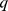 we have so far studied). Finally, we note that the three most frequent phenotypes are found relatively faster because they satisfy 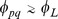. As expected, for this larger system the fraction of phenotypes for which this holds is lower than for the random GP map with smaller 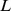.

Overall, the evolutionary dynamics of this rather complex RNA system resembles that of the much simpler random GP map. Most importantly, the discovery times vary over many orders of magnitude. More precisely, as long as 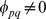, 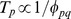 for both the monomorphic and polymorphic regimes: Phenotypic bias leads to a simple, systematic ordering in the discovery of novel phenotypes.

### The arrival of the frequent

The many orders of magnitude difference in the arrival rate of variation between phenotypes should have many important implications for evolutionary dynamics. Consider for example the situation where the population has equilibrated to a phenotype 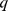, which was the fitness peak, when subsequently the environment changes so that a different phenotype 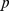 has a higher fitness 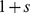. In order to fix, the alternative phenotype must first be found. If the time-scale 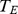 on which the environment changes again is much longer than 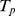 then it likely that the population will discover and fix 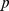. However, if 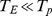, then a new phenotype 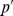 may become more fit before 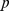 has time to fix. 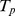 can vary over many orders of magnitude, so many potentially highly adaptive phenotypes may satisfy 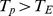 and thus never be found.

Consider also the situation where two phenotypes 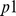 and 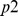 are both more fit than 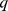 after an environmental change. If 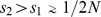, then in a standard population genetics picture, we would expect 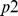 to fix rather than 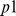 as long as 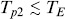. However, this argument ignores the rate at which variation arises. If, for example, 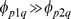, then 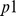 may fix well before 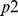 is discovered and fixes.

**Figure 3:**
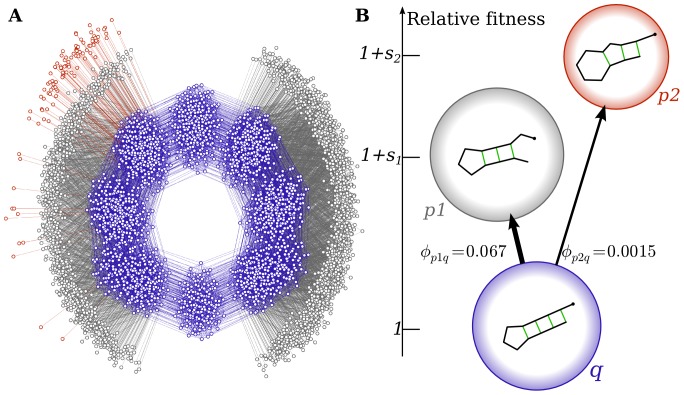
Interconnections of neutral spaces in RNA influence evolutionary trajectories. *A*) 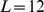 RNA neutral component for phenotype 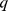 with 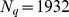 genotypes (drawn in blue). Lines depict single mutations to itself, or to two alternative phenotypes 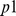 (grey) and 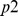 (red). The genotypes were ordered using the Fruchterman-Reingold algorithm [30]. *B*) Illustration of the fitness landscape.

To illustrate this effect, we study the 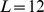 RNA system depicted in Figure 3, where the source neutral space has 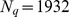 genotypes, while the two target phenotypes have 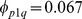 and 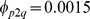, so 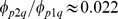, a relatively modest ratio compared to the what could be found from e.g. Fig. 2. For this particular system 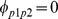: there are no direct single mutation connections between the two target phenotypes – 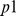 and 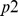 are distinct peaks of the fitness landscape.

We simulated a population of 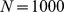 individuals with fixed 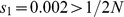, but with varying ratios 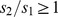. The population begins on phenotype 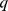 and evolves until 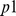 or 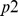 fixes.

**Figure 4:**
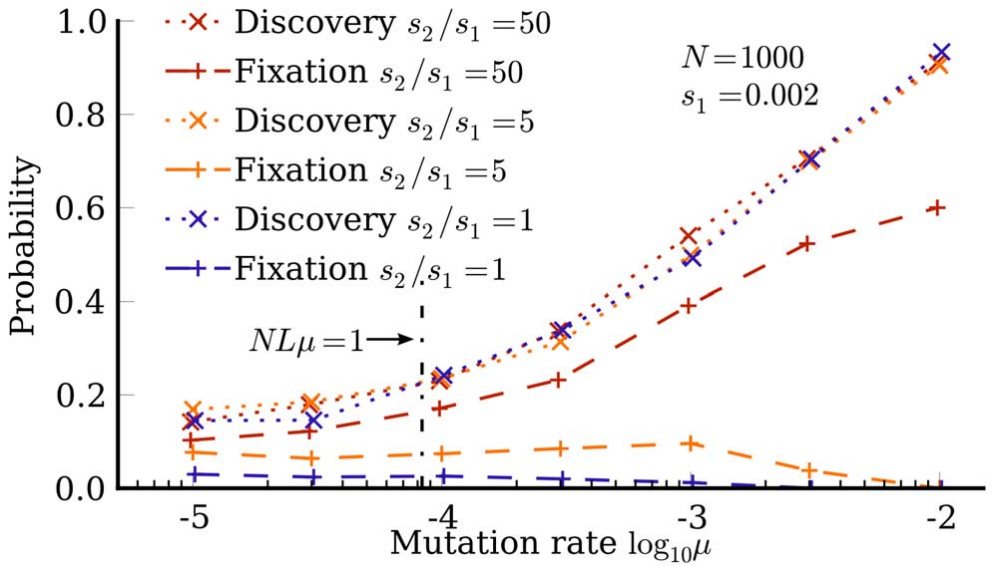
The arrival of the frequent. Probability that phenotype 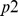 is discovered (dotted lines) or is fixed (dashed lines) as a function of mutation rate 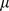 for different relative selection coefficients 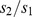 for 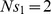. The probability that 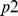 is discovered is independent of relative fitness (within statistical simulation errors). Phenotype 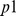 is much more likely to fix than phenotype 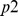, even when the latter is much more fit, due to an “arrival of the frequent” phenomenon.

Results are shown in Figure 4. As the mutation rate increases and the system moves from the monomorphic to polymorphic regime, the probability that 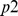 is discovered at least once increases (and is largely independent of fitness). Nevertheless, phenotype 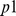 is discovered much earlier and also much more often because 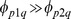. Furthermore, in the monomorphic regime where 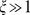 the population remains on a single genotype 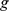 much longer than it takes to explore all the neighbours. Thus if 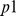 is accessible from 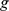, then 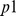 is likely arise repeatedly in relatively quick succession (in “bursts”). This effect, which arises naturally in our microscopic model [24], can significantly enhance the probability of fixation over that predicted by origin-fixation models [25] which ignore the discreteness of the source neutral space.

Overall, our simulations show how the more frequent phenotype 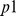 can fix at the expense of the more fit phenotype 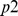. Given the many orders of magnitude difference possible between the 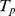, such an “arrival of the frequent” effect may prevent the arrival of the fittest: If a highly beneficial phenotype is never discovered, a much less adaptive but easily accessible phenotype may go to fixation instead.

Finally, phenotype 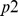 is significantly less mutationally robust than 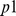 (more frequent phenotypes are typically more robust [13], [16]), and so once discovered, produces deleterious mutants at a higher rate, making it harder for 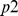 to fix at higher mutations rates, a phenomenon known as “survival of the flattest” [26], observed here for the lower ratios 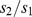 at higher 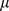. Thus both the “arrival of the frequent” and the “survival of the flattest” mitigate against the fixation of phenotypes with lower frequency 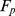, even if their fitness is much higher.

We note that differences in neutral network size have traditionally also been taken into account in terms of free fitness [27], which – in analogy with free energy in statistical physics [28] – incorporates an entropy-like component to account for mutational effects such as genetic drift and mutational robustness. This picture provides a theoretical foundation for the “survival of the flattest” [26] effect we observe at high mutation rates in Figure 4. However, the “arrival of the frequent” effect is fundamentally different because it does not rely on mutation-selection balance and quasi-equilibrium or steady-state assumptions like free-fitness theory does. Rather, it reflects the strongly non-equilibrium effect that 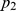 is rarely or never found. In the example above, the difference in discovery times between 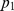 and 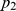 is rather modest, and so at large enough mutation rates 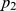 is found fairly regularly and free-fitness could be used to analyse results in that regime. But as can be seen for instance in Figure 2 for 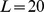 RNA, differences in discovery times can vary over many more orders of magnitude than is the case for our particular example, so that in practice highly adaptive yet rare phenotypes may not be discovered at all, even on very long timescales.

## Discussion

Mutations provide the fuel for natural selection. Based on this principle, we have presented a detailed model of evolutionary dynamics that focuses on a microscopic description of the outcome of mutations. The phenotypic effect of mutations is mediated by the genotype-phenotype (GP) map which is therefore a crucial ingredient. As outlined in the introduction, several generic features are shared by many different example maps, independent of model details. Here we mainly focussed on the fact that these mapping are highly *biased*: Some phenotypes are realised by orders of magnitude more genotypes than most other phenotypes.

Our calculations for a simplified random mapping and for the more complex RNA secondary structure model predict that the large bias observed in the GP maps translates into a similar order of magnitude variation in the median discovery times 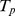 for a range of population genetic parameters. For both maps the local frequencies 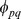 (which predict discovery times) are a good predictor for the discovery times 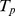. For the random GP map 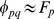. For RNA this relationship provides a rough first order estimate, but the local frequencies can also deviate strongly, especially when 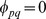, which can occur even when the global frequency 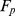 is large. For both maps the strong bias in the GP map leads to a systematic *ordering* of the median discovery times of alternative phenotypes, an effect that we postulate may hold for other GP maps as well.

In light of the simplicity of our mean-field approximation, its success in predicting the first-discovery time 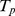 (cf. Figure 2) is rather striking. In the random GP map, the excellent agreement probably arises because all genotypes in the source neutral space are similar in the sense that they have the same probability distribution to have a certain mutational neighbourhood. There are static fluctuations because the number of neighbours is less than the number of states with 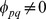. But while these fluctuations have an effect on processes like fixation, they average out over the many runs used to find the mean or median 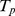. By contrast, in the RNA GP map mutational neighbourhoods of adjacent genotypes are often correlated [13], [23] so that a single neutral mutation does not completely re-shuffle the accessible phenotypes (as the mean-field assumption would assume). This effect explains why the value of the exploration parameter 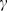 we obtain by fitting is below the value suggested by our mean-field model, and also why we still observe around 1 order of magnitude variation in 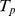 for very similar values of 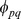 (see Figure 2). Despite such correlations (which we postulate may occur in other realistic GP maps), rare phenotypes (low 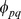) remain hard to find; the strong phenotypic bias in the RNA GP map provides a good a posteriori justification for our mean-field calculations: The many orders of magnitude range in 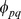 dominates the scale of the phenotype discovery times.

The large differences we observe in the rate with which potential variation appears should have many consequences for evolutionary dynamics. There is of course a long history of invoking processes that impose directionality on the pathways available for evolutionary exploration (see ref. [29] for a recent discussion). Here, by solving microscopic population genetic models, we show in detail just how strong these orienting processes can be. Other authors have also pointed out how evolution may favour phenotypes with large neutral networks for RNA, see e.g. refs. [5], [22]. Similar points have been made for protein models [12]. Consider, for example, our 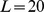 RNA system. Despite its rather modest size, we find 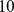 orders of magnitude difference between the discovery times of frequent and rare phenotypes. These differences should be even more pronounced for larger 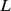. In nature, selectable RNA phenotypes are of course characterised by more than just their secondary structure, and evolutionary processes don't always work at constant 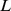. Nevertheless, it is hard to see how such enormous variations in 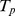 would not persist in some form in much more sophisticated treatments of biological RNA. Similar arguments can be made for the other GP maps we listed above. More generally we emphasise that including the GP map in population genetic calculations may be of importance to a wide range of evolutionary questions.

We explicitly showed how phenotypes with a high local frequency can fix at the expense of locally rare phenotypes, even if the latter have much higher fitness. Taken together, these arguments suggest that the vast majority of possible phenotypes may never be found, and thus never fix, even though they may globally be the most fit: Evolutionary search is deeply non-ergodic. When Hugo de Vries was advocating for the importance of mutations in evolution, he famously said “Natural selection may explain the survival of the fittest, but it cannot explain the arrival of the fittest” [2]. Here we argue that the fittest may never arrive. Instead evolutionary dynamics can be dominated by the “arrival of the frequent”.

## Methods

### Simulations

In the dynamic simulations, all 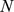 individuals of the population are initially assigned to a single random genotype in the source neutral space. Then the population evolves for 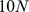 generations to reach a steady-state dispersal on the neutral space before measurements are started.

### RNA

Secondary structures for RNA were predicted from sequence using the Vienna package [15], version 1.8.5 with all parameters set to their default values.

## Supporting Information

Appendix S1 (PDF)

Figure S1Static properties of the random GP map. *A*) Global phenotypes frequencies. In addition to the distribution of frequencies 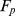 used in our simulations (orange), the diagram also shows the frequencies of RNA secondary structures at 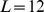, obtained by exhaustive enumeration using the Vienna package, Version 1.8.5 with all parameters set to their default values [15]. *B*) Comparison of global frequencies 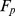 and local frequencies 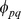 for the source neutral space 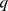 with rank 1. The robustness of phenotype 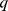 (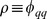) is marked in green; alternative phenotypes (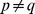) are shown in light blue. The dashed line marks the equality of global and local frequency 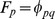. The relative size of deviations becomes more severe as 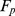 becomes small: The less genotypes map into 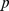, the less will frozen fluctuations in the GP map average out.(TIF)

Figure S2
**Total number of mutants per phenotype in different dynamic settings.** The diagram shows the total number of mutants 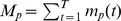 carrying phenotype 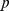 that were produced during a total of 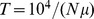 generations of simulation under the random GP map. Dots show the average over 100 simulations, error bars show the standard deviation. The dashed lines correspond to the mean-field theory 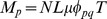 that follows directly from Eq. (3). In panels B and D, the populations are in the highly polymorphic regime (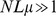) and hence evolve towards greater robustness [19] so that the total number of non-neutral mutants is reduced.(TIF)

Figure S3
**Scaling of** 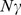 **with population dynamic parameters.** The diagram shows the dependence of 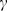 on: *A*) mutation rate 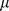, *B*) population size 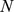 and *C*) number of mutants per generation 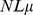. Note that the y-axis has been scaled by population size 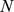.(TIF)

Figure S4
**Scaling of** 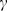 **with population dynamic parameters.** The diagram shows the dependence of 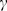 on: *A*) mutation rate 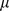, *B*) population size 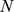 and *C*) number of mutants per generation 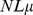. In contrast to Figure S3, the y-axis shows 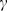 without any scaling factors.(TIF)

Figure S5
**Phenotypic bias for RNA secondary structures of length** 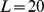 **.** *A*) Global phenotype frequencies 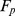 for all 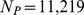 secondary structures. It required about 1 CPU-year on typical present-day hardware to fold all 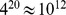 sequences once using the fold-routine of the Vienna package [15], version 1.8.5 with all default parameters. *B*–*D*) Local phenotype frequencies 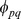 around 3 neutral spaces. An example sequence and its secondary structure is given in each panel; starting from this sequence, the 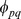 can be obtained exactly by tracing out all possible neutral mutations and counting how often each phenotype is produced. *Insets*: Comparison of global and local frequencies. Accessible phenotypes (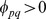) are drawn in blue, inaccessible phenotypes (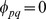) are shown in orange and the phenotype corresponding to the neutral space itself is shown in green (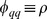). The dashed line marks the equality of local and global frequencies 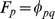 and the dotted line indicates the minimal (non-zero) local frequency 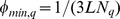, corresponding to only a single mutation away from one of the 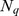 genotypes in the neutral space. Inaccessible phenotypes with very small global frequencies are omitted for clarity. Note that all these phenotypes are relatively rare ones when compared to Fig. 2b.(TIF)

Figure S6
**Predictions based on global frequency.** The diagram shows the same median discovery times of alternative RNA secondary structures that are displayed in Figure 2c, but here as a function of the phenotypes' global frequencies 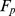 rather than their local frequencies 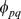. The different colors indicate: Accessible phenotypes that are typically discovered within the simulation time (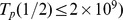, 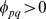, light blue); accessible phenotypes that are typically not discovered (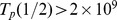, 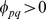, dark blue); inaccessible phenotypes that are typically discovered (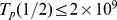, 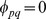, orange); inaccessible phenotypes that are typically not discovered (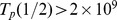, 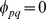, red). The lines correspond to the prediction for 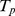 based on global rather than local frequencies: 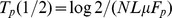 (cf. Eq. (4)), dashed) and 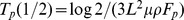 (cf. Eq. (7)). In contrast to the predictions based on the local frequencies 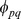 in Figure 2c, we note the following: 1) Several phenotypes arise even earlier than predicted by the analogue of the polymorphic limit (points below dashed line). 2) Many phenotypes are not discovered even though other phenotypes of comparable (and even much lower) frequency do arise during the simulation. 3) 4 of the most frequent, but locally inaccessible phenotypes are discovered on a time-scale when double mutations become relevant (orange dots; since 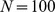 and 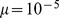, double mutants occur on the timescale 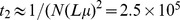, so if double mutations were to lead to globally random phenotypes, we expect phenotypes with 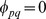 to be discovered around 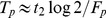.)(TIF)
