# Supplementary material for: The Arrival of the Frequent: How Bias in Genotype-Phenotype Maps Can Steer Populations to Local Optima
Source: PLoS One. 2014 Feb 5;9(2):e86635. doi: 10.1371/journal.pone.0086635 (PMC3914804; doi:10.1371/journal.pone.0086635)
Supplement: Appendix S1 — (PDF) [file pone.0086635.s001.pdf]

Supporting Information Appendix 1

The dependence of γ on population dynamic parameters

For the limiting regimes of polymorphic and monomorphic populations (with $NL\mu \gg 1$ and $NL\mu \ll 1$, respectively), we have given complete predictions for the first discovery time of an alternative phenotype p depending on N, μ and ϕ_{pq} in Equations (4) and (7) Based on these results, we argued that between these regimes, there should be an interpolating factor γ such that a full expression for the first discovery time is

$$T_p(\alpha) = \frac{-\log(1-\alpha)}{N\gamma L\mu\phi_{pq}} \tag{1}$$

where all symbols take the same meaning as in the main text.

Based on the results in the paper, we predict the following limiting behaviour for γ: 1) $\gamma \approx 1$ in the large genome limit $L \gg N$; 2) $\gamma \approx 1$ in polymorphic populations ($NL\mu \gg 1$); 3) $\gamma \to (K-1)L\rho_q$ in large, monomorphic populations ($NL\mu \ll 1$ but $N \gg L$).

Calculating γ explicitly is beyond the scope of this work. Instead, we study the behaviour of γ numerically through extensive simulations under the random GP map, as outlined above. To this end, we performed 100 simulations for many combinations (N,μ) spanning several orders of magnitude for each parameter. The value of γ is calculated from the observed median discovery times $\hat{T}_p$ by a least-squares fit to Equation (1) (with $\alpha = 1/2$, as we consider the median discovery times):

$$\gamma = \frac{\log 2 \sum_p \phi_{pq}\hat{T}_p}{NL\mu \sum_p (\phi_{pq}\hat{T}_p)^2} \tag{2}$$

Figure S3 shows the simulation results, with γ multiplied by population size N to facilitate interpretation. As expected, when $NL\mu \gg 1$, we see that $N\gamma$ approaches N, that is $\gamma \to 1$: In polymorphic populations, there is little loss of diversity under genetic drift. By contrast, as $NL\mu$ becomes small, we see that $N\gamma$ tends to $(K-1)L\rho$, showing that in such monomorphic populations, the localization in genotype space slows down the discovery of alternative phenotypes. Finally, we see that in the large genome limit ($N = 10$, which is smaller than $L = 12$), γ is roughly independent of μ and is just below unity, as we would expect based on our theoretical arguments. The general scaling behaviour of γ for intermediate values of $NL\mu$ is complex and shows no simple dependencies on the dynamic parameter N and μ (cf. Figures S3 and S4).
