# Supplementary figures and images for: The Arrival of the Frequent: How Bias in Genotype-Phenotype Maps Can Steer Populations to Local Optima
Source: PLoS One. 2014 Feb 5;9(2):e86635. doi: 10.1371/journal.pone.0086635 (PMC3914804; doi:10.1371/journal.pone.0086635)

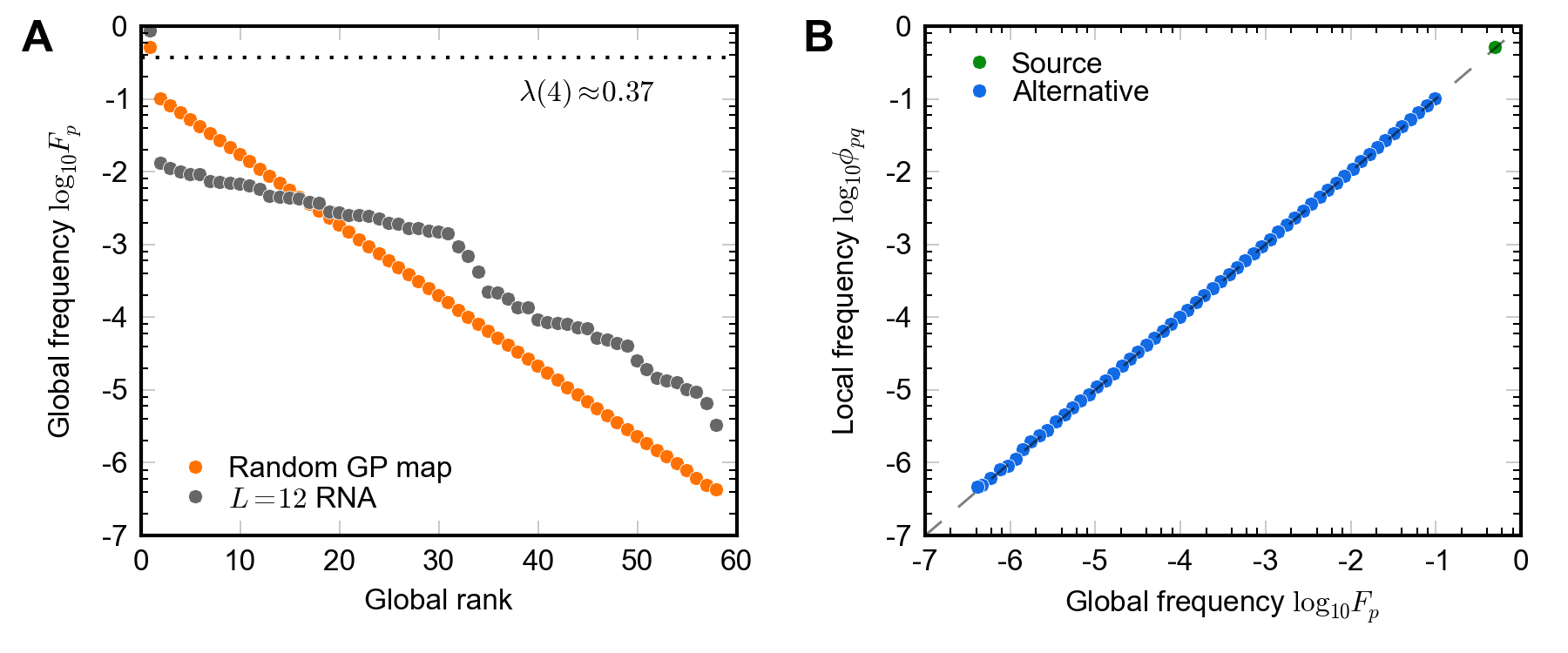

Supplement: Figure S1 — Static properties of the random GP map. A) Global phenotypes frequencies. In addition to the distribution of frequencies used in our simulations (orange), the diagram also shows the frequencies of RNA secondary structures at , obtained by exhaustive enumeration using the Vienna package, Version 1.8.5 with all parameters set to their default values [15]. B) Comparison of global frequencies and local frequencies for the source neutral space with rank 1. The robustness of phenotype () is marked in green; alternative phenotypes () are shown in light blue. The dashed line marks the equality of global and local frequency . The relative size of deviations becomes more severe as becomes small: The less genotypes map into , the less will frozen fluctuations in the GP map average out. (TIF) [file pone.0086635.s002.tif]

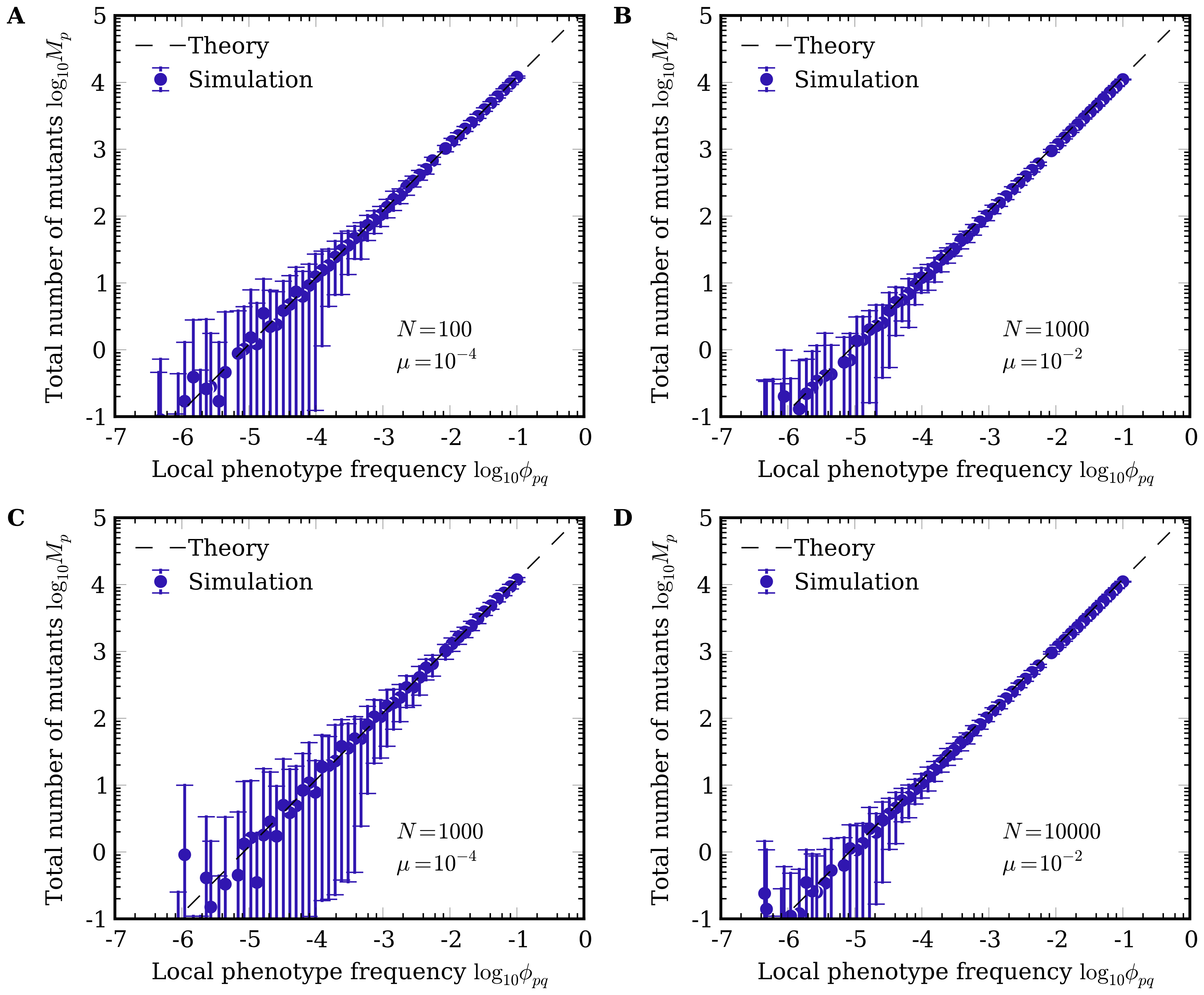

Supplement: Figure S2 — Total number of mutants per phenotype in different dynamic settings. The diagram shows the total number of mutants carrying phenotype that were produced during a total of generations of simulation under the random GP map. Dots show the average over 100 simulations, error bars show the standard deviation. The dashed lines correspond to the mean-field theory that follows directly from Eq. (3). In panels B and D, the populations are in the highly polymorphic regime () and hence evolve towards greater robustness [19] so that the total number of non-neutral mutants is reduced. (TIF) [file pone.0086635.s003.tif]

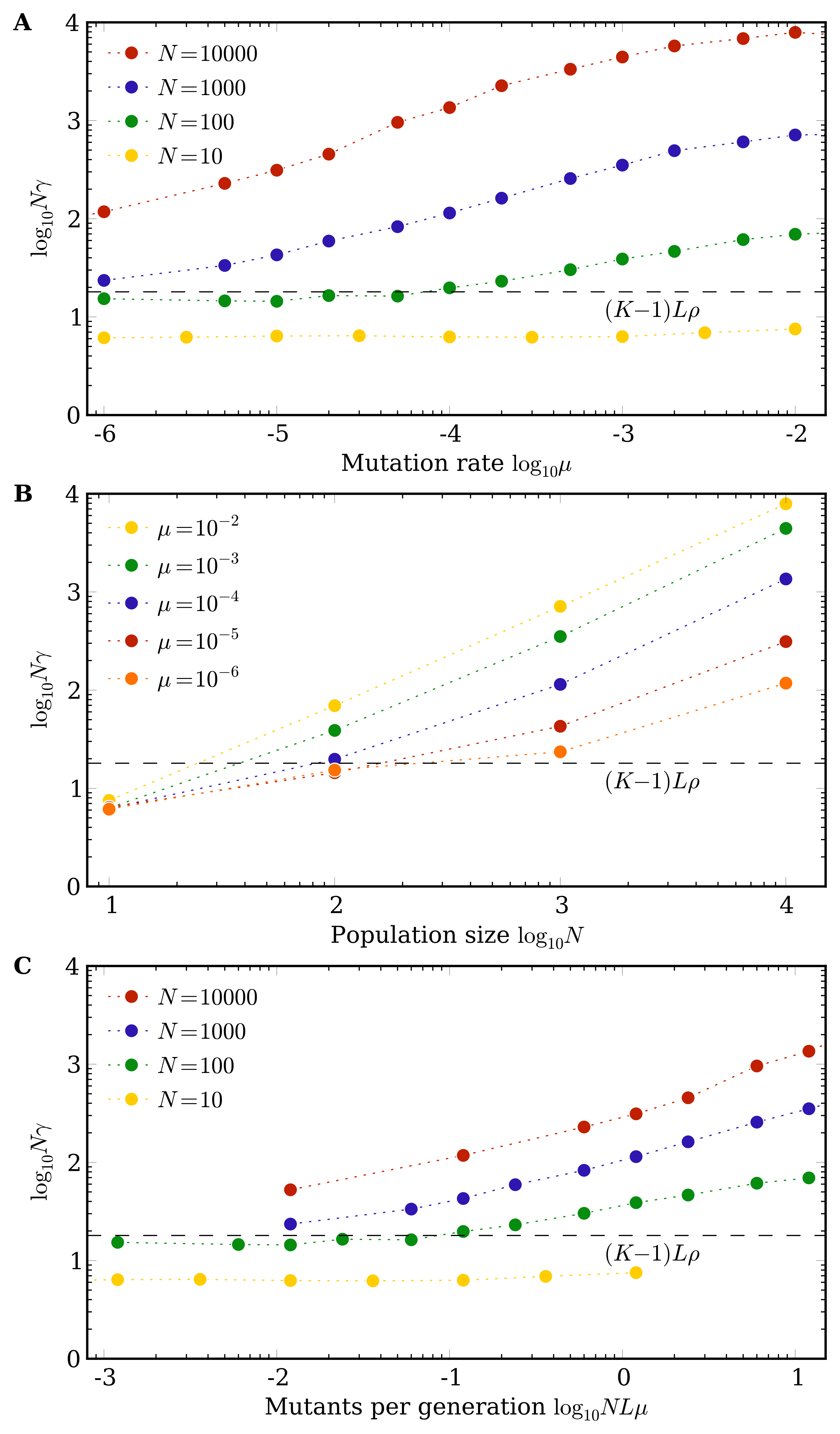

Supplement: Figure S3 — Scaling of with population dynamic parameters. The diagram shows the dependence of on: A) mutation rate , B) population size and C) number of mutants per generation . Note that the y-axis has been scaled by population size . (TIF) [file pone.0086635.s004.tif]

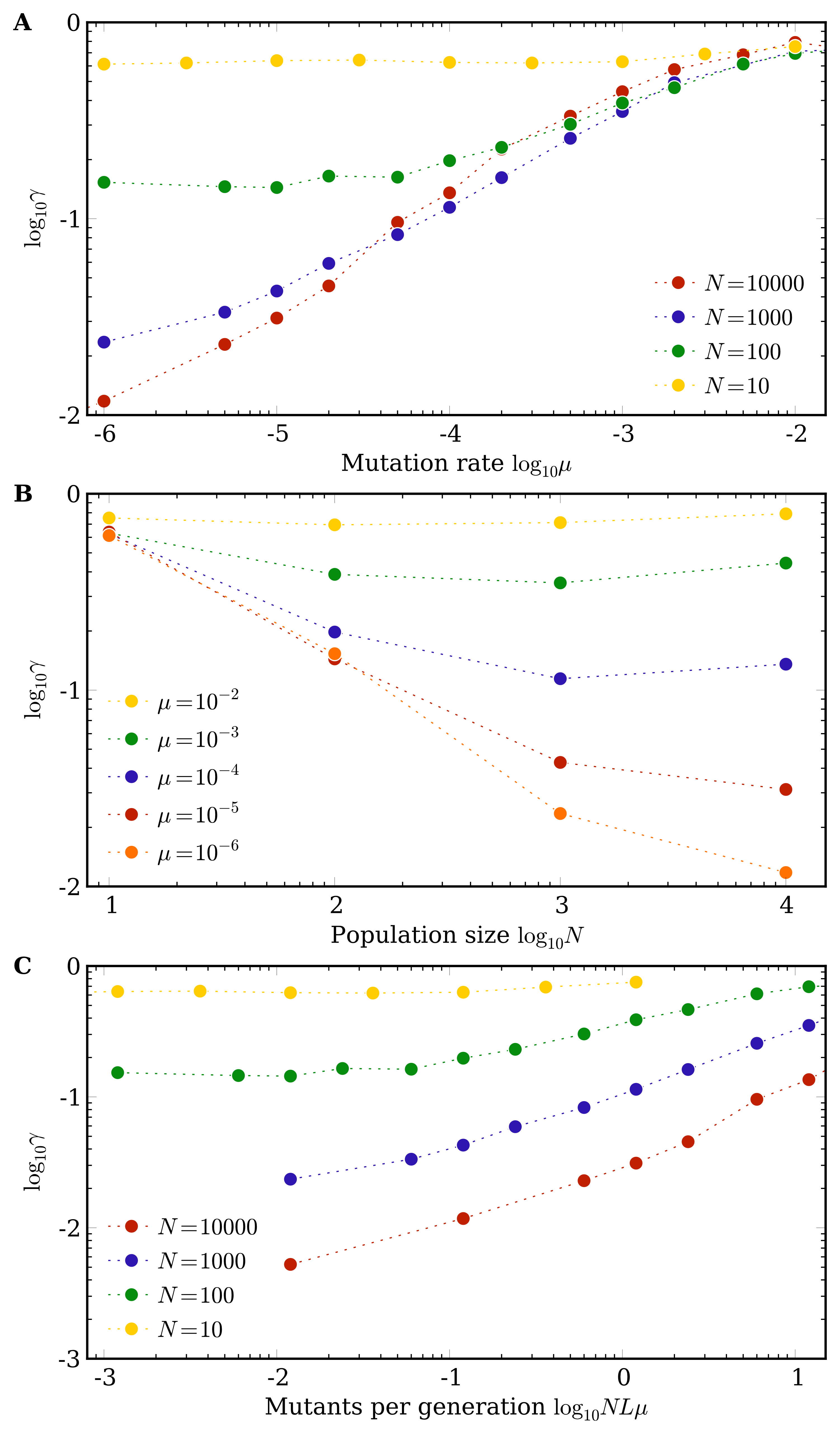

Supplement: Figure S4 — Scaling of with population dynamic parameters. The diagram shows the dependence of on: A) mutation rate , B) population size and C) number of mutants per generation . In contrast to Figure S3, the y-axis shows without any scaling factors. (TIF) [file pone.0086635.s005.tif]

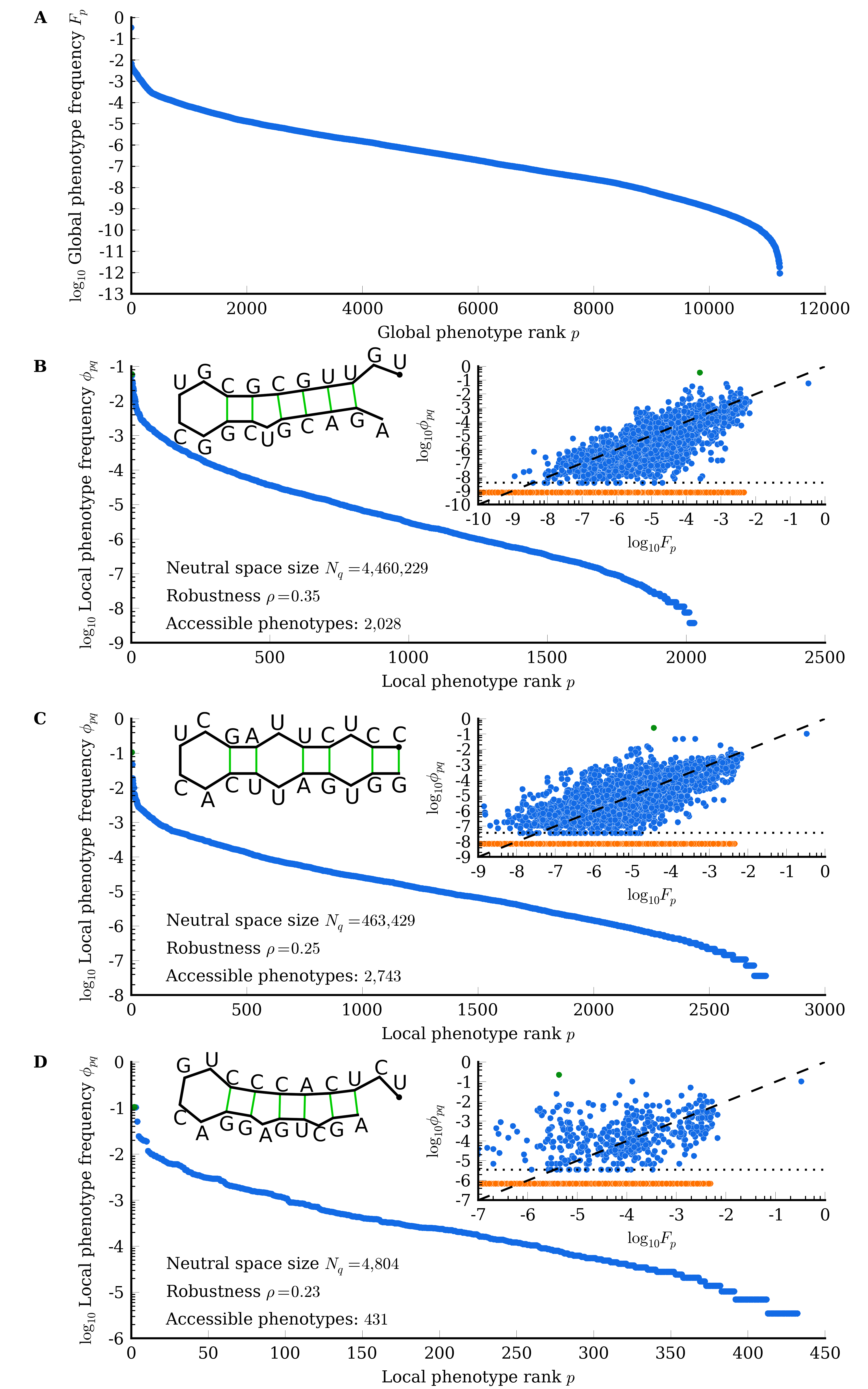

Supplement: Figure S5 — Phenotypic bias for RNA secondary structures of length . A) Global phenotype frequencies for all secondary structures. It required about 1 CPU-year on typical present-day hardware to fold all sequences once using the fold-routine of the Vienna package [15], version 1.8.5 with all default parameters. B–D) Local phenotype frequencies around 3 neutral spaces. An example sequence and its secondary structure is given in each panel; starting from this sequence, the can be obtained exactly by tracing out all possible neutral mutations and counting how often each phenotype is produced. Insets: Comparison of global and local frequencies. Accessible phenotypes () are drawn in blue, inaccessible phenotypes () are shown in orange and the phenotype corresponding to the neutral space itself is shown in green (). The dashed line marks the equality of local and global frequencies and the dotted line indicates the minimal (non-zero) local frequency , corresponding to only a single mutation away from one of the genotypes in the neutral space. Inaccessible phenotypes with very small global frequencies are omitted for clarity. Note that all these phenotypes are relatively rare ones when compared to Fig. 2b. (TIF) [file pone.0086635.s006.tif]

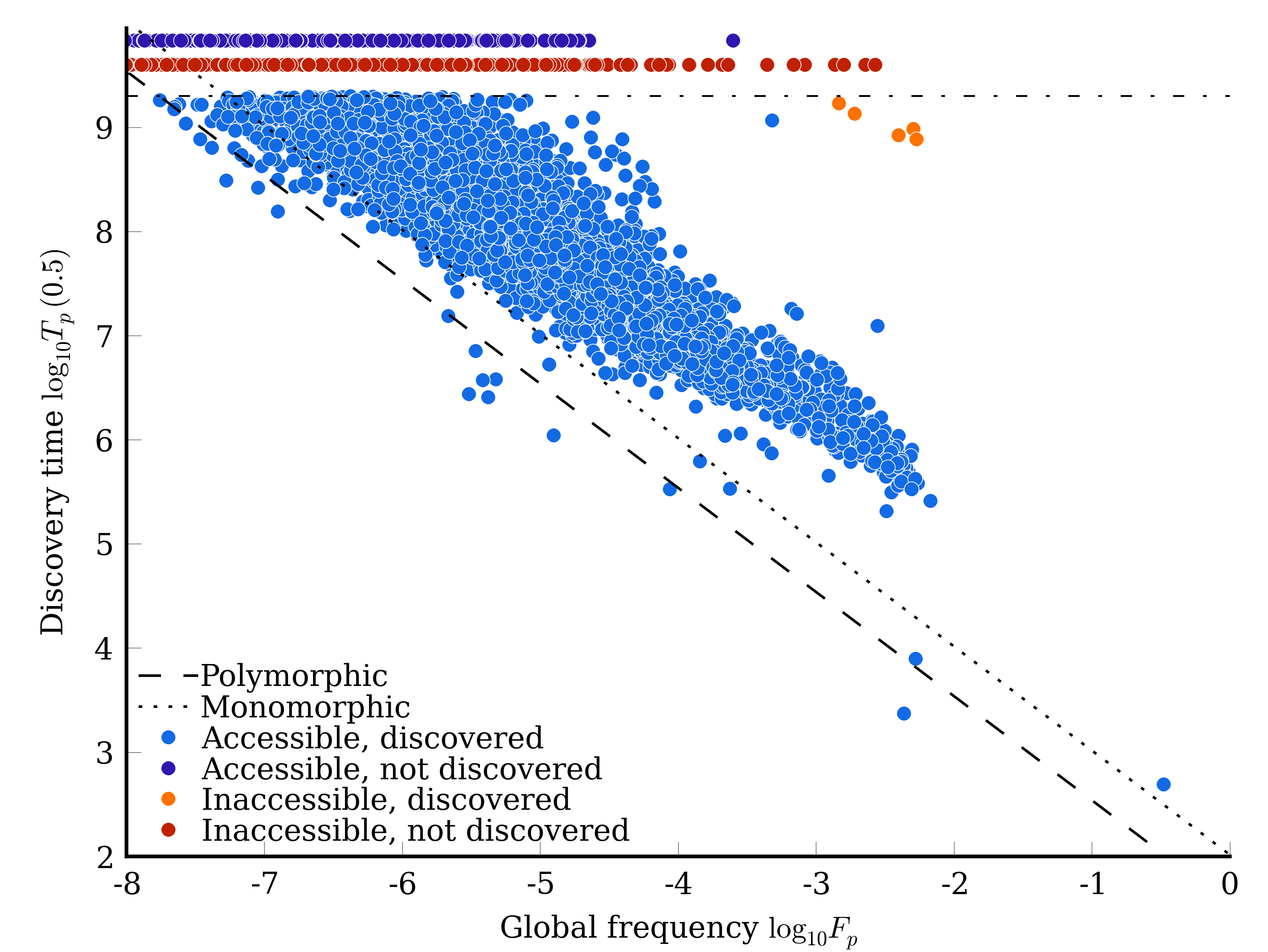

Supplement: Figure S6 — Predictions based on global frequency. The diagram shows the same median discovery times of alternative RNA secondary structures that are displayed in Figure 2c, but here as a function of the phenotypes' global frequencies rather than their local frequencies . The different colors indicate: Accessible phenotypes that are typically discovered within the simulation time (, , light blue); accessible phenotypes that are typically not discovered (, , dark blue); inaccessible phenotypes that are typically discovered (, , orange); inaccessible phenotypes that are typically not discovered (, , red). The lines correspond to the prediction for based on global rather than local frequencies: (cf. Eq. (4)), dashed) and (cf. Eq. (7)). In contrast to the predictions based on the local frequencies in Figure 2c, we note the following: 1) Several phenotypes arise even earlier than predicted by the analogue of the polymorphic limit (points below dashed line). 2) Many phenotypes are not discovered even though other phenotypes of comparable (and even much lower) frequency do arise during the simulation. 3) 4 of the most frequent, but locally inaccessible phenotypes are discovered on a time-scale when double mutations become relevant (orange dots; since and , double mutants occur on the timescale , so if double mutations were to lead to globally random phenotypes, we expect phenotypes with to be discovered around .) (TIF) [file pone.0086635.s007.tif]
